# In vivo assessment of mitochondrial capacity using NIRS in locomotor muscles of young and elderly males with similar physical activity levels

**DOI:** 10.1007/s11357-019-00145-4

**Published:** 2019-12-19

**Authors:** Bart Lagerwaard, Arie G. Nieuwenhuizen, Vincent C. J. de Boer, Jaap Keijer

**Affiliations:** 1 Human and Animal Physiology, Wageningen University and Research, PO Box 338, 6700 AH Wageningen, The Netherlands; 2 TI Food and Nutrition, PO Box 557, 6700 AN Wageningen, The Netherlands

**Keywords:** Near-infrared spectroscopy, Sarcopenia, Reperfusion, Oxidative metabolism

## Abstract

Mitochondrial capacity is pivotal to skeletal muscle function and is suggested to decline with age. However, there is large heterogeneity in current data, possibly due to effect modifiers such as physical activity, sex and muscle group. Yet, few studies have compared multiple muscle groups in different age groups with comparable physical activity levels. Here, we newly used near-infrared spectroscopy (NIRS) to characterise mitochondrial capacity in three different locomotor muscles in young (19–25 year) and older (65–71 year), healthy males with similar physical activity levels. Mitochondrial capacity and reperfusion after arterial occlusion was measured in the vastus lateralis (VL), the gastrocnemius (GA) and the tibialis anterior (TA). Physical activity was verified using accelerometry and was not different between the age groups (404.3 ± 214.9 vs 494.9 ± 187.0 activity kcal per day, *p* = 0.16). Mitochondrial capacity was significantly lower in older males in the GA and VL, but not in the TA (*p* = 0.048, *p* = 0.036 and *p* = 0.64, respectively). Reperfusion rate was not significantly different for the GA (*p* = 0.55), but was significantly faster in the TA and VL in the young group compared to the older group (*p* = 0.0094 and *p* = 0.039, respectively). In conclusion, we identified distinct modes of mitochondrial ageing in different locomotor muscles in a young and older population with similar physical activity patterns. Furthermore, we show that NIRS is suitable for relatively easy application in ageing research and can reveal novel insights into mitochondrial functioning with age.

## Introduction

Ageing is associated with a decline in skeletal muscle mass and strength, also known as sarcopenia (Morley et al. 2001). Sarcopenia is thought to be mediated in part by a decline in skeletal muscle mitochondrial capacity, as both the amount of mitochondria and their capacity to generate energy decrease with age in the muscle, resulting in a reduced endurance capacity (Welle et al. 2003; Short et al. 2005; Marzetti et al. 2013). Improving or sustaining muscle mitochondrial capacity could delay the age-related decline in endurance capacity, ultimately retaining physical function and improving quality of life (Lanza et al. 2008; Coen et al. 2013).

Due to the pivotal function of mitochondria in the process of ageing, it is essential to routinely and robustly assess mitochondrial capacity. In vivo 31-phosphorus magnetic resonance spectroscopy (^31^P-MRS) and near-infrared spectroscopy (NIRS) are existing techniques that can be applied to assess mitochondrial capacity in vivo. Typically, for both techniques, assessment of mitochondrial capacity involves the recovery of muscle homeostasis after exercise; however, ^31^P-MRS measures the recovery of phosphocreatine (PCr), whereas NIRS measures the recovery of muscle oxygen consumption (mV̇O_2_) as a parameter for mitochondrial capacity (Kemp et al. 2015; Grassi and Quaresima 2016). NIRS makes use of the difference in light absorption in the near-infrared region of oxygenated (O_2_Hb) and deoxygenated haemoglobin and myoglobin (HHb) and can therefore be used to monitor muscle oxygenation. Combining NIRS with arterial occlusions is used to measure mV̇O_2_ in the muscle in vivo (Hamaoka et al. 1996)*.* The mV̇O_2_ recovery kinetics after exercise follow a monoexponential function of which the rate constant is used as a measure for mitochondrial capacity, as better-functioning mitochondria will recover mV̇O_2_ faster (Motobe et al. 2004). This application of NIRS correlated well to ^31^P-MRS measurements of PCr recovery and ex vivo high-resolution respirometry (Ryan et al. 2013, 2014b). Although ^31^P-MRS is more widely used, NIRS offers advantages over ^31^P-MRS due to its higher mobility, relatively low costs and higher throughput, making NIRS more suitable for routine measurements to, for example, study the effect of age on muscle mitochondrial capacity. Despite its easier applicability, NIRS has not been used to assess the effects of age on muscle mitochondrial capacity in locomotor muscles.

Ex vivo respiratory analysis of muscle biopsies taken from the vastus lateralis (VL) show a consistent, negative effect of age on muscle oxidative capacity (Short et al. 2005; Irving et al. 2015; Porter et al. 2015; Lalia et al. 2017). Yet, ^31^P-MRS analysis of different muscle types report a heterogeneous effect of age on PCr recovery (Fitzgerald et al. 2016). In the VL, most studies showed a negative effect of age on PCr recovery (Conley et al. 2000; Johannsen et al. 2012; Larsen et al. 2012; Choi et al. 2016), but on other locomotor muscles, such as the gastrocnemius muscle (GA) and tibialis anterior (TA), this effect was not observed (Chilibeck et al. 1998; Wray et al. 2009; Larsen et al. 2012; Tevald et al. 2014; Hart et al. 2015). However, some studies do find a negative effect of age on PCr recovery in the GA (McCully et al. 1993; Waters et al. 2003; Layec et al. 2013) and it has been suggested that the conflicting results could arise from the use of different populations with different physical activity levels. Physical activity has a positive effect on muscle oxidative capacity (Tonkonogi and Sahlin 2002), and is thought to be able to protect from, or at least mitigate, the deteriorating effect of age (Lanza et al. 2008; Larsen et al. 2012). Yet, physical activity is documented to decrease with advancing age (Troiano et al. 2008) and therefore isolating the effect of age on mitochondrial capacity is challenging, as this effect is often entangled with a decrease in physical activity. Therefore, controlling for the confounding effects of physical activity is essential, if not a requisite, in studies looking into the effect of age on mitochondrial capacity.

Since it is unclear how mitochondrial capacity is affected in different muscle types with ageing, we aimed to profile mitochondrial capacity using NIRS in three different muscle types, i.e. the GA, TA and VL in young and older healthy males. These muscles serve an important function during locomotion and are accessible by NIRS due to their superficial position. To negate the effect of physical activity, subjects with similar self-reported physical activity patterns were included in the study and this was verified using accelerometry measurements. We hypothesised a lower mitochondrial capacity in the GA and the VL and an unaffected or higher mitochondrial capacity in the TA.

## Materials and methods

### Subjects

Healthy males between the age of 19–25 (young) and 65–71 (older) years were recruited from the local population. Low to moderately physical active individuals were recruited using a self-reported exercise frequency of 1–2 h of structured physical activity per week or a Baecke habitual physical activity score between 7 and 10 points (Baecke et al. 1982). Older individuals were not physically impaired as determined using the short performance battery test (SPPB) with a minimum score of 11 (Guralnik et al. 1994). None of the subjects identified as regular smoker used recreational drugs during the study or reported recent use of performance-enhancing drugs or supplements. Subjects were non-anaemic (haemoglobin concentration > 8.0 mmol/L), verified by using HemoCue Hb 201 microcuvette (HemoCue AB, Sweden). None of the subjects had health concerns regarding respiratory or metabolic disease. One elderly subject used cholesterol-lowering medication, one used diuretics and one used both cholesterol-lowering medication and a diuretic.

### Experimental protocol

The subjects refrained from heavy physical exercise 48 h prior to testing and from any exercise and alcohol consumption 24 h prior to testing. Maximal voluntary contraction (MVC) hand grip strength was measured using a Jamar Hydraulic Hand Dynamometer (Performance Health, IL, USA). The dominant and the non-dominant arm were assessed three times while seated upright at a table. The subjects performed the measurement while the dynamometer was resting on the table with the elbow at a 90° angle. Highest value out of three 5-s isometric contractions was set as MVC. Body fat percentage was determined according to the four-site method by Durnin-Womersley using skinfold calliper (Harpenden, UK) measurements of the triceps, biceps, sub scapula and supra iliac. Furthermore, skinfold between NIRS receiver and transmitter was measured on the GA, TA and VL.

### NIRS measurements

Deoxyhaemoglobin (HHb) and oxyhaemoglobin (O_2_Hb) were continuously measured using a PortaMon wireless, dual-wavelength system (760 and 850 nm; PortaMon, Artinis Medical Systems, Netherlands). The optode distance (distance between emitter and receiver) of 40 mm was used for analysis. Data were collected at 10 Hz via bluetooth using Oxysoft software (Artinis Medical Systems). The NIRS probe was placed longitudinally 10–15 cm distal to the knee cap on the GA and TA and 15 cm proximal to the kneecap on the VL. To secure the probe and protect it from environmental light, the probe was tightly taped to the skin. To measure oxygen consumption, a blood pressure cuff (Hokanson SC5 and SC12; D.E. Hokanson Inc., Bellevue, WA) was placed proximally of the probe just below the knee joint or as high up on the thigh as possible. The cuff was powered and controlled by a rapid cuff inflator system (Hokanson E20 and AG101 Air source; D.E. Hokanson Inc.) set to a pressure of 250 mmHg. Post-exercise muscle oxygen consumption recovery was assessed similar to previously published protocols (Ryan et al. 2013). In summary, the protocol consists of three 30-s rest measurements of basal oxygen consumption. To calibrate the signal between individuals, the minimal-oxygenation of the tissue underneath the probe was then determined after 30 s of exercise followed by a 4 min occlusion until signal plateaus. The hyperaemic response after the cuff was released was considered maximal oxygenation. Recovery oxygen consumption was measured immediately after 30-s exercise until 50% of oxygenation signal, using a series of transient occlusions (5 × 5 s on/5 s off, 5 × 7 s on/7 s off, 10 × 10 s on/10 s off). Recovery measurements were performed twice with 2-min rest in between tests. Thirty-second exercise is defined as 30-s plantar flexion using a rubber resistance band for GA (until 50% of oxygenation signal), 30 s of dorsiflexion using a rubber resistance band for TA (until 50% of oxygenation signal) and 30 s of twitch electrical stimulation (biphasic, duration interval 200/50 μs, 4 Hz) was used for the VL. Four electrodes (Compex, USA) were placed on the skin, proximal and distal to the NIRS and connected to a Compex Pro – THETA electrical stimulator (Chattanooga, USA). Current intensity was adjusted individually to a maximal tolerable level.

### Reperfusion measurements

Reperfusion rate was measured using the recovery of the O_2_Hb NIRS signal after the 4-min arterial occlusion performed during the NIRS protocol and was defined as the half-life in seconds to reach maximal oxygenation. Maximal oxygenation was defined as the plateau in O_2_Hb NIRS signal, i.e. O_2_Hb-signal did not increase for 10 s. The subject was in supine position with slight elevation of the upper body and instructed to sustain from any movement during reperfusion.

### Analysis of muscle oxygen consumption data

NIRS data were blinded and analysed using Matlab-based (The Mathworks, MA, USA) analysis software (NIRS_UGA, GA, USA). Data were analysed as 100% of maximal oxygenation. mV̇O_2_ was calculated during every arterial occlusion using the slope of the change in HHb and O_2_Hb for 3 s for the 5-s occlusions, for 5 s for the 7-s occlusions, 7 s for the 10-s occlusions and 15 s for the basal measurements. A blood volume correction factor was used for each data point (Ryan et al. 2012) to correct for retributions of blood distally from the cuff. mV̇O_2_ recovery measurements post-exercise were fitted to a monoexponential curve:

$$ y\ (t)=\mathrm{End}-\Delta  \times {e}^{-k\bullet t} $$

where *y* represents the mV̇O_2_ during the arterial occlusions, End being the mV̇O_2_ immediately after the cessation of exercise, delta (∆) being the difference between mV̇O_2_ after exercise and mV̇O_2_ during rest, *k* being the rate constant expressed in time units and *t* being time. Rate constants of duplicates were averaged. Rate constants calculated from curve fitting with *R*^2^ < 0.95 were excluded from analysis for GA and TA as a measure of poor data quality. For VL a curve fitting until *R*^2^ < 0.90 was accepted due to lower muscle activation using electrical stimulation.

### Accelerometry

Subjects were instructed to wear triaxial accelerometer (wGT3X-BT, Actigraph, USA) for seven consecutive days using an elastic band at the waist of the non-dominant leg. The accelerometer was worn during all activities, excluding showering, swimming and sleeping. Wear time was manually verified using daily diaries on shower and bed times. Counts were sampled at 30 Hz and stored in 60 s epochs to determine counts per minute (CPM). Two weekend days and three weekdays were used for data analysis. Percentage of wear time in sedentary (SPA), light (LPA) and moderate to vigorous physical activity (MVPA) was determined using the cutoffs provided by Troiano et al., being 0–99 CPM, 100–2019 CPM and 2020–∞ CPM respectively (Troiano et al. 2008). Activity kcals per day were calculated using the counts from all axis according to the Freedom VM3 equation (Sasaki et al. 2011).

### Statistical analyses

Data were presented as mean ± SD. Statistical analyses were performed using GraphPad Prism v.5 (GraphPad Software, CA, USA). Means between the two groups were compared using a Students unpaired *t* test. Normality was tested using Shapiro-Wilk normality test. Correlations between variables were calculated using regression analysis. Means between three muscles were compared using one-way ANOVA with Tukey’s multiple comparison test. Significance was accepted at *p* < 0.05.

## Results

### Subject characteristics

All subjects completed all tests without any contra-indications. Most physical characteristics were similar, yet the older males were significantly heavier than the young individuals and had a significantly higher fat mass (Table 1). Fat-free mass and MVC were not significantly different between the two groups. Skinfolds on measurements sites were similar for VL and TA but were slightly lower for old compared to young on the GA. All older individuals had a SPPB score of 11 or higher indicating normal physical function in this group.

**Table 1:** Physical characteristics of the subjects

	Young (*n* = 20)	Older (n = 20)	*p* value
Age (years)	22 ± 2.0	69 ± 1.9	*< 0.0001*
BMI (kg/m^2^)	22.6 ± 1.9	25.4 ± 1.7	*< 0.0001*
Body fat (%)	15.3 ± 3.1	25.1 ± 3.9	*< 0.0001*
Fat free mass (kg)	63.5 ± 6.9	61.0 ± 5.0	0.200
MVC dominant arm (kg)	54 ± 7	50 ± 7	0.125
Skinfolds (mm)			
Vastus lateralis	10.4 ± 2.7	9.1 ± 2.7	0.157
Gastrocnemius	10.4 ± 2.8	7.6 ± 2.4	*0.002*
Tibialis anterior	9.3 ± 3.1	8.5 ± 3.7	0.566
Haemoglobin (mmol/L)	9.4 ± 0.4	9.1 ± 0.7	0.163
SPPB score		11.65 ± 0.5	
Ethnicity	Caucasian (18), Asian (1), Indo-Pacific (1)	Caucasian (20)	
Physical activity			
Baecke questionnaire score	8.3 ± 0.8	8.7 ± 0.9	0.161
SPA (%)	79.1 ± 7.3	75.4 ± 5.8	0.084
LPA (%)	15.5 ± 3.4	19.6 ± 5.4	*0.006*
MVPA (%)	4.4 ± 2.2	5.0 ± 2.2	0.405
Activity kcal (kcal/day)	404.3 ± 214.9	494.9 ± 187.0	0.1629
Wear time (h/day)	14.6 ± 1.1	15.4 ± 0.8	*0.011*

Data is presented as mean ± standard deviation. MVC is maximum voluntary contraction. Time spent in sedentary physical activity (SP), light physical activity (LPA), and moderate-to-vigorous physical activity (MVPA) are expressed as a percentage of total wear time. MET is metabolic equivalent of task. TEE is total energy expenditure. PAEE is physical activity energy expenditure. PAL is physical activity level. SPPB is short physical performance battery

### Physical activity

**Fig. 1:**
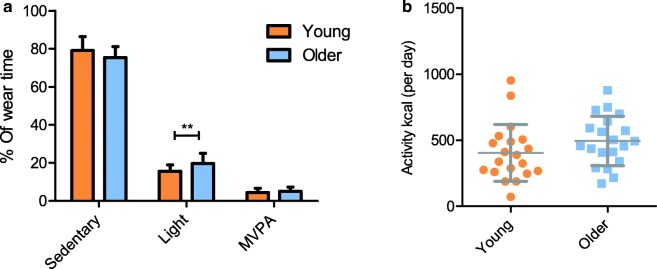
Percentage of wear time spent in sedentary, light and moderate-to-vigorous physical activity (**a**) and calculated activity kcal (**b**) for young and older group calculated from accelerometry data. Values are mean ± SD. **p < 0.005

Reported physical activity using the Beacke questionnaire was not significantly different between the two groups (Table 1). Measured PA using accelerometer showed that MVPA was not significantly different between the young and older individuals; however, older individuals spent significantly more time in LPA (*p* = 0.006) at the expense of time spent in SPA (*p* = 0.084; Fig. 1a). Activity kcal per day was not different between the groups (*p* = 0.1629; Fig. 1b) and wear time was significantly higher in the older group compared to the young group.

### Mitochondrial capacity in the gastrocnemius, tibialis anterior and vastus lateralis

**Fig. 2:**
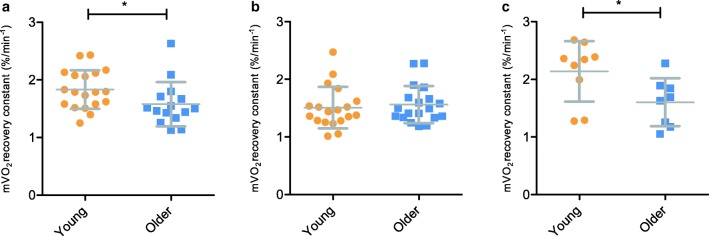
Recovery constants derived from monoexponential curve fits of mV̇O_2_ recovery from NIRS measurements after 30 s of plantar flexion exercise in gastrocnemius (**a**), 30s of dorsiflexion exercise in tibialis anterior (**b**) and 30 s of electrical stimulation in vastus lateralis (**c**). Values are mean ± SD. *p < 0.05

Mitochondrial capacity was measured using repeated occlusions after a short exercise protocol in the GA, TA and VL. Mitochondrial capacity was significantly different between young and old group for GA (*p* = 0.048; Fig. 2a) and VL (*p* = 0.036; Fig. 2c), but not for the TA (*p* = 0.64; Fig. 2b). When comparing mitochondrial capacity between the three muscles, there was a significant higher mitochondrial capacity in VL compared to TA (*F* = 5.33, *p* = 0.006). Two data sets for TA and six data sets for GA were excluded due to *r*^2^ < 0.95. Twenty-four data sets for the VL were excluded due to insufficient muscle activation using electrostimulation or a *r*^2^ < 0.90.

### Reperfusion rate in gastrocnemius, tibialis anterior and vastus lateralis

**Fig. 3:**
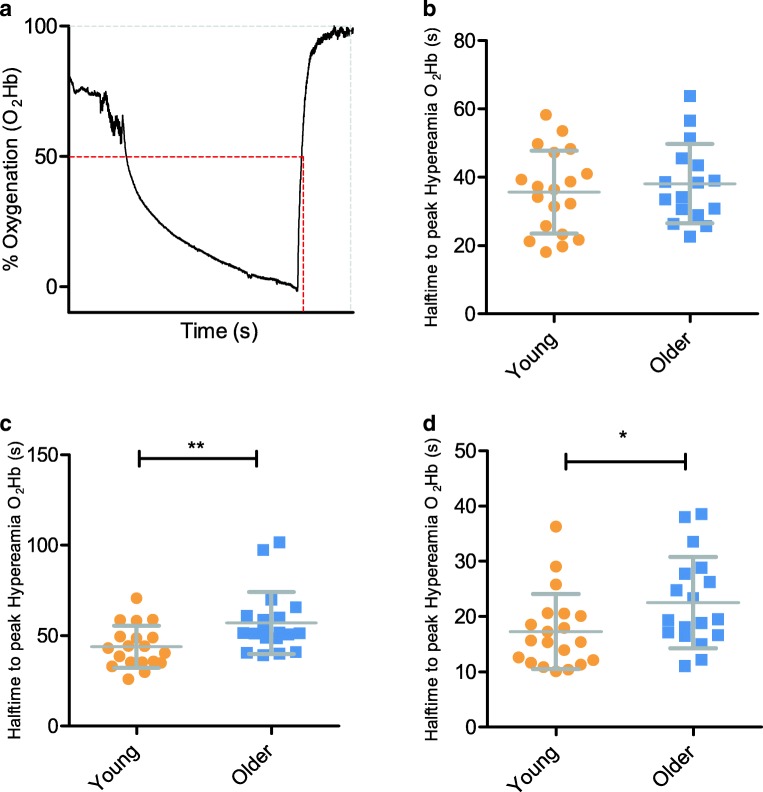
Representative plot of NIRS measurements of O_2_Hb during 4 min occlusion and reperfusion measurements. Red dotted line represents time to reach 50% oxygenation (halftime to peak hyperaemia) as a measure for reperfusion rate (**a**). Reperfusion was measured in the gastrocnemius (**b**), tibialis anterior (**c**) and vastus lateralis (**d**). Values are mean ± SD. *p < 0.05 **p < 0.005

Reperfusion rate was measured after a 4-min occlusion and defined using the half lifetime at 50% maximal oxygenation (Fig. 3a). Reperfusion rate was not significantly different in the GA (*p* = 0.55; Fig. 3b) but was significantly faster in the TA and VL in the young group compared to the older group (*p* = 0.0094 and *p* = 0.039 respectively; Fig. 3c, d).

### Associations between mitochondrial capacity and physical activity

**Fig. 4:**
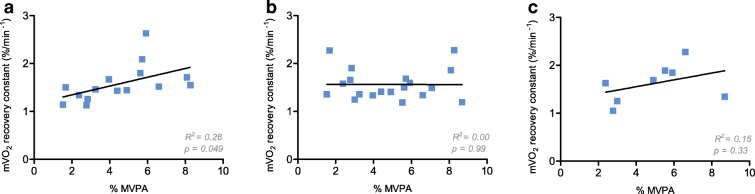
Correlation of recovery constants derived from monoexponential curve fits of mV̇O_2_ recovery from NIRS measurements after 30 s of plantar flexion exercise in gastrocnemius (**a**, *n* = 15), 30 s of dorsiflexion exercise in tibialis anterior (**b**, *n* = 19) and 30 s of electrical stimulation in vastus lateralis (**c**, *n* = 8) and percentage of wear time spent in moderate-to-vigorous physical activity (%MVPA) for the older group

The mitochondrial capacity of the GA was significantly correlated to time spent in MVPA in the older group (*R*^2^ = 0.27, *p* = 0.048), but this was not the case not for the TA and VL (*R*^2^ = 0.0, *p* = 0.99 and *R*^2^ = 0.15, *p* = 0.34 respectively; Fig. 4). In the young group, MVPA was not correlated to mitochondrial capacity for the three muscles.

## Discussion

The primary objective of this study was to determine the effect of age on mitochondrial capacity in young and older healthy males in three different locomotor muscles. Due to the similar moderate-to-vigorous physical activity levels between the older and young individuals in our study, it was possible to assess the effect of age on muscle mitochondrial capacity, independent from the effect of physical activity. Ageing negatively affected mV̇O_2_ recovery in the GA and VL, but not in the TA, showing that the age-driven decline in mitochondrial capacity is muscle specific. Furthermore, reperfusion rate after a 4-min occlusion was decreased with age in the VL and TA, but not in the GA, showing that it is important to consider parameters of vascularisation in muscle mitochondrial measurements.

We are the first to measure the effect of age on mV̇O_2_ recovery in three different locomotor muscle using NIRS. Other research primarily used ^31^P-MRS to measure mitochondrial capacity in vivo and, although ^31^P-MRS and NIRS are based on the same underlying assumption of post-exercise recovery of metabolism and NIRS is correlated with ^31^P-MRS (Ryan et al. 2013), these techniques do measure distinct physiological mechanisms (i.e. recovery of mV̇O_2_ or PCr, respectively). Since a decrease in coupling of mitochondrial oxygen consumption, or phosphorus to oxygen ratio (P/O), has been reported with ageing (Amara et al. 2007), one could hypothesise that, if uncoupling is an important feature in mitochondrial ageing, more oxygen would be needed for the recovery of the same amount of PCr in old compared to young, directly affecting measurements of mV̇O_2_ recovery, yet only indirectly affecting PCr recovery. Since we observed similar ageing effects in the current study compared to existing in vivo literature (Kent and Fitzgerald 2016), we conclude that NIRS is applicable in ageing research to faithfully measure mitochondrial capacity, which is corroborated by a NIRS study performed in young and old individuals, but was not controlled for physical activity (Chung et al. 2018).

### Ageing and muscle mitochondrial capacity

In the GA, a slower mV̇O_2_ recovery with age was observed and this was negatively correlated with %MVPA in the older group. Additionally, we observed a correlation between time spent in MVPA and mV̇O2 recovery. Therefore, it could be that higher levels of physical activity are required to preserve mitochondrial capacity of the GA with age. Yet, the effect of age on mitochondrial capacity in the GA is debated. Some studies observed a negative effect of age on PCr recovery in the GA (McCully et al. 1993; Waters et al. 2003; Layec et al. 2013), whereas others did not observe this (Waters et al. 2003; Wray et al. 2009; Tevald et al. 2014; Hart et al. 2015). Although it is challenging to compare the literature due to differences in study populations with regard to sex and physical activity level, the results of the current study seem unique in its kind, since a decrease in mV̇O_2_ recovery with age was observed despite controlling for physical activity. Interestingly, one other study that measured PCr recovery in older individuals with decreased PA compared to young did not observe an age effect (Tevald et al. 2014). Yet, the aforementioned study included both males and females and had lower sample size for males only. While not all studies found an effect of age on mitochondrial capacity, according to our data, some degree mitochondrial ageing in the GA seems to be inevitable, yet having a higher MVPA is associated with mitigating this unfavourable effect.

In the VL, a 25% slower mV̇O_2_ recovery in older individuals was observed, reflecting a decrease in mitochondrial capacity with age. This finding is in agreement with studies measuring PCr recovery in the VL (Conley et al. 2000; Johannsen et al. 2012; Larsen et al. 2012; Choi et al. 2016; Adelnia et al. 2019) and with studies measuring ex vivo oxygen consumption using high-resolution respirometry (Porter et al. 2015; Distefano et al. 2018). Specifically, Larsen et al. found a 23% decrease in PCr recovery in older adults compared to younger adults in a similar-aged and physical activity-matched population (Larsen et al. 2012). Therefore, the VL seems to be particularly affected by ageing, even when physical activity levels were maintained. In fact, we did not observe a correlation between %MVPA and mV̇O_2_ recovery in the VL, suggesting that increased levels of physical activity do not preserve the VL from an age-driven decline in mitochondrial capacity. However, this result should be interpreted with care, due to the low number of measurements that were included in the correlation. Furthermore, inactive or sedentary older individuals do show a lower mitochondrial capacity than their more active counterparts, indicating that a protective effect of physical activity on the VL may not be excluded (Larsen et al. 2012; Distefano et al. 2018). The lower number of measurements in VL were the result of insufficient activation of the muscle during the NIRS protocol. A previous study reported sufficient muscle activation using a similar electrical stimulation (Brizendine et al. 2013). Yet, for half of the participants, we were unable to produce sufficient muscle activation, and thus measure increased muscle oxygen consumption and its recovery. Possibly, a 30-s isometric contraction could have been used for muscle activation, as this has been shown to be a good alternative for electrical stimulation (Ryan et al. 2014a).

While the GA and VL are affected by age in the current study, the mV̇O_2_ recovery in the TA is not significantly different between the young and the older individuals. This confirms consensus in literature that age does not affect mitochondrial capacity in this muscle (Kent-Braun and Ng 2000; Lanza et al. 2007; Christie et al. 2014). A possible explanation for this distinct effect could be attributed to intrinsic characteristics of the TA, such as its fibre type composition. The TA has a higher proportion of oxidative type I fibres than the GA and VL (Jakobsson et al. 1988) and it has been suggested that ageing less severely effects mitochondria in predominantly type I muscle fibres due to a mild induction of uncoupling, possibly reducing the production of reactive oxygen species and consequent damage (Amara et al. 2007). This could explain why the TA is protected from the age-driven decline in mV̇O_2_ recovery. Yet, this hypothesis is not supported by research in mice, where the most oxidative muscle, the soleus, was shown to be most negatively affected by age (Picard et al. 2011). Moreover, it cannot be excluded that extrinsic factors also play a role in the resistance against the age-driven decline in mV̇O_2_ recovery. The TA is unique in its higher activation during locomotion with advancing age (Jakobsson et al. 1988), whereas the demand on other muscles, such as the VL, is thought to decrease with age (Hortobágyi and DeVita 2000; Tirosh and Sparrow 2005), perhaps exerting muscle-specific (de)training-like adaptations that can affect mitochondrial capacity. Alternatively, the TA may intrinsically be less susceptible to the effects of physical activity. Indeed, the effect of a 5-week bed rest did not affect TA thickness, while the VL and GA thickness were significantly reduced after this period, showing that decreased activity did not induce muscle atrophy in TA as much as in other muscles (de Boer et al. 2008). Moreover, the TA has a lower association with MVPA compared to the VL in young and elderly subjects (Larsen et al. 2009, 2012), further supporting that TA is less affected by the levels of physical activity. Therefore, differences in fibre type, different activation patterns or susceptibility to (de)training-like adaptations could explain why the TA seems to be less susceptible to the age-driven decline in mitochondrial capacity.

Analysing mV̇O_2_ recovery of both the young and the older group together, there was a significantly higher mitochondrial capacity in the VL compared to the TA. This difference in mV̇O_2_ recovery between muscles has been previously shown in elderly males and in young adults (Larsen et al. 2009, 2012). Also, it has been reported that the GA has a higher mitochondrial capacity than the TA in elderly women (Tevald et al. 2014), which we also observed in our data when just GA and TA were compared directly. In the TA, the majority of fibres is classified as oxidative type I fibres, whereas the VL and GA have more even distribution between type I and type II fibres (Edgerton et al. 1975; Henriksson-Larsen et al. 1983; Jakobsson et al. 1988). Therefore, it seems that the percentage of type I fibres does not predispose a higher mitochondrial capacity, at least not measured using these in vivo techniques. Besides, in the current study, we observed that the muscle with the highest reported proportion of type I fibres is also most resistant to ageing. However, this is speculative in its nature because no fibre typing was done in the tissue underneath the NIRS probe and it is unknown what is the effect of fibre type on mVO_2_ recovery measured using NIRS. Yet, it does advocate that the metabolic properties of these muscles are influenced by their intrinsic characteristics and that exploring these muscle-specific effects could help to elucidate ageing mechanisms.

### Ageing and muscle reperfusion rate

Ageing has been associated with a decrease in vasodilation and microvascular function in the muscle (Tonson et al. 2017). Recovery of mV̇O_2_ or recovery of PCr is only a measure for mitochondrial capacity if oxygen availability is not limited during the recovery period. In the current study, we measured reperfusion rate as the return to maximal oxygenation after a 4-min arterial occlusion. This was significantly lower in the TA and VL in the older group compared to the young group, while no difference in reperfusion rate was observed in the GA. Although there was an age-related decline in reperfusion in the TA, this did not result in an age-related decline in mV̇O_2_ recovery. This suggest that the muscle reperfusion rate was not limiting for mV̇O_2_ recovery in our study. This notion is further supported by our results in GA, where reperfusion rates are not different between the two age groups, but a difference in mV̇O_2_ recovery was observed. In contrast, a decrease in end-exercise perfusion rate was reported in a study by Wray et al., which was not accompanied by a decrease PCr recovery (Wray et al. 2009). However, differences in physical activity were not accounted for in this study. With exercise and physical activity being a known inducer of angiogenesis, this result could partly be explained by differences in physical activity. The effect of age on reperfusion rate was not observed by Hart et al., who did neither observe a decrease in oxygen delivery nor a decrease in PCr recovery between young and older adults in the GA (Hart et al. 2015).

On the other hand, for the VL, both reperfusion rate and mitochondrial capacity were decreased in the older males in the current study. Therefore, we cannot be certain that the mV̇O_2_ recovery was not negatively affected by the decrease reperfusion rate with age in this muscle. However, using the current protocol, muscle oxygenation during testing does not fall below 50% of one’s maximal oxygenation. Therefore, it is unlikely that oxygen availability was a limiting factor during mV̇O_2_ recovery, attributing the age-related decline in mV̇O_2_ recovery to decreased mitochondrial capacity. Moreover, a decrease in perfusion rate can be compensated by an increased oxygen diffusion to the muscle tissue, ultimately not lowering oxygen availability at the level of mitochondria.

Nevertheless, our data do not show that oxygen availability or reperfusion rate is per definition not a limiting factor for physical functioning with advancing age, as it can be still be limiting during maximal or prolonged periods of exercise. This is supported by recent research, which reported that ageing was associated with a decrease in resting muscle perfusion in middle-aged and elderly adults. Resting muscle perfusion rates were negatively associated with muscle PCr recovery and whole-body oxidative capacity, suggesting that changes in reperfusion could affect physical functioning with advancing age (Adelnia et al. 2019). However, in that study, the population was not matched for physical activity, making it again difficult to draw conclusions from the results. Therefore, measuring reperfusion of the muscle should be considered good practice, because impaired reperfusion could violate the underlying assumptions of the mitochondrial capacity measurement. To our knowledge, we are the first to measure the effect of age on reperfusion rate in three locomotors muscles using NIRS and an advantage of the current NIRS protocol is that it allows assessment of mitochondrial capacity and reperfusion in the same measurement.

## Conclusion and further perspectives

The current study provides evidence for distinct modes of mitochondrial ageing in different locomotor muscles in a relatively large population of physical activity-matched young and older males. We identified that the TA, but not the VL and GA, was resistant to an age-driven decline in mitochondrial ageing. This exemplifies the limitations of generalising results obtained in one type of muscle to other muscles and underlines the importance of controlling for important effect modifiers such as sex, physical activity or even muscle activity assessed using electromyography. Being the first to use NIRS to study the effect of age on mitochondrial capacity, we show that NIRS is suitable for application in ageing research. Application of this non-invasive technique could accelerate research in this field, including studies on the clinical efficacy of interventions aiming to improve either vascular function (e.g. Kiss et al. 2019) or mitochondrial function (e.g. Nacarelli et al. 2018) in an elderly population.

An advantage of using in vivo techniques over ex vivo measurements is the possibility to study muscle mitochondrial capacity in the intact system, with a physiological oxygen pressure and the dynamic interplay between oxygen delivery, diffusion and consumption. However, ex vivo respirometry measurements on muscle biopsies more directly assess mitochondrial capacity and can provide mechanistic insights into muscle ageing. Since the VL is mostly used for taking biopsies, there is a gap in current literature for the ex vivo mitochondrial capacity other muscles than the VL, possibly presenting a biased view on mitochondrial ageing in the muscle. Identifying the molecular signatures of age-unaffected muscles, such as the TA, could shine new lights upon mechanisms underlying muscle ageing. This could provide leads for pharmacological or tailored exercise interventions to counteract the decline mitochondrial capacity with advancing ageing, potentially retaining physical function and improving quality of life.
